# Chlorotoxin-conjugated onconase as a potential anti-glioma drug

**DOI:** 10.3892/ol.2014.2835

**Published:** 2014-12-29

**Authors:** XIAOMIN WANG, ZHANYUN GUO

**Affiliations:** Institute of Protein Research, College of Life Sciences and Technology, Tongji University, Shanghai 200092, P.R. China

**Keywords:** chlorotoxin, glioma, onconase, chemical conjugation, targeted therapy

## Abstract

Gliomas are rarely curable malignant brain tumors arising from normal glial cells. The scorpion-derived small peptide, chlorotoxin (CTX), can selectively bind malignant gliomas. In the present study, a CTX-conjugated onconase (Onc), a small cytotoxic ribonuclease, was prepared as a potential anti-glioma drug. In this conjugate, recombinant CTX was covalently linked with recombinant Onc by reversible disulfide linkage. The chemically conjugated CTX-Onc showed much higher cytotoxicity to the cultured glioma U251 and SHG-44 cells than the physical mixture of CTX and Onc (CTX + Onc). In the nude mouse models bearing subcutaneous U251 or SHG-44 tumors, the CTX-Onc conjugate also showed improved anti-tumor effects than the CTX + Onc control. These results suggested that the reversible chemical-conjugated CTX promoted the tumor targeting of Onc, and thus the present CTX-Onc conjugate could be further developed as a potential targeted anti-glioma drug.

## Introduction

Gliomas account for 80% of malignant brain tumors and are rarely curable (1–3). The traditional treatments, including surgical excision, chemotherapy and radiotherapy, have limited effects on gliomas. Targeted therapy is a promising approach for cancer treatment, but identification of the tumor-specific target remains difficult (4–6). In recent years, a scorpion-derived polypeptide chlorotoxin (CTX) was found to selectively bind malignant gliomas (7,8) mediated by the cell surface matrix metalloproteinase-2 (MMP-2) and annexin-2, whose expression is increased in gliomas (9,10). CTX was first isolated from the scorpion venom in 1993 and is composed of 36 amino acids and four disulfide bonds (11). In previous years, the glioma-binding properties of CTX have been extensively investigated and CTX conjugates have been developed for the treatment and diagnosis of gliomas and other malignant tumors (12–21). The radioactive iodine-131-labeled CTX has been designated as an orphan drug for the treatment of malignant glioma and melanoma by the US Food and Drug Administration, and other fluorescent dye- or nanoparticle-labeled CTXs also show great potential for glioma diagnosis and treatment (22).

Onconase (Onc) is a small RNase that was first isolated from oocytes of the northern leopard frog (*Rana pipiens*) in 1988 (23). Onc is composed of 104 amino acids and four disulfide bonds (24), and shares a similar tertiary structure with other members of the ribonuclease A superfamily (25). Subsequent to its identification, Onc was revealed to exhibit anti-tumor properties, as it can enter the tumor cells, degrade RNA and cause cell death (26–29); thus, Onc is being developed as an anti-tumor drug for several types of malignant tumors (30–33). As CTX exhibits glioma-targeting properties and Onc exhibits cytotoxicity, in the present study a CTX-conjugated Onc (CTX-Onc) was prepared for the analysis of its anti-glioma effects on cultured glioma cells and on a mouse model.

## Materials and methods

### Preparation of the recombinant CTX and Onc

The recombinant CTX was prepared from a GST-6′His-CTX precursor by enzymatic cleavage and *in vitro* refolding, according to our previously described procedure (34). The recombinant Onc was prepared through heterologous expression of an N-terminally 6xHis-tagged Onc (6xHis-Onc) precursor by enzymatic cleavage, N-terminal cyclization and *in vitro* refolding, according to our previously described procedure (35).

### Chemical conjugation of the recombinant CTX with the recombinant Onc

To label CTX with N-Succinimidyl 3-(2-pyrdydithio)propionate (SPDP; Thermo Fisher Scientific, Rockford, IL, USA), the lyophilized recombinant CTX was dissolved in 1.0 mM aqueous HCl (Sinopharm, Beijing, China)and the SPDP reagent in dimethylsulfoxide (DMSO). To start the reaction, they were mixed together at equal concentrations (400 μm each) in the reaction buffer (50 mM phosphate and 150 mM NaCl; pH 8.0) and incubated at room temperature for 1 h. Subsequent to the reaction mixture being acidified to pH 3.0 by trifluoroacetic acid (Merck KGaA, Darmstadt, Germany), high performance liquid chromatography (Agilent Technologies, Santa Clara, CA, USA) was applied to the mixture, according to our previously described chromatography methods (35). The labeled CTX fractions were eluted from a C18 reverse-phase column (Agilent Technologies) by an acidic acetonitrile gradient, manually collected, and lyophilized.

To label Onc with 2-iminothiolane (Thermo Fisher Scientific), the recombinant Onc was dissolved in 1.0 mM aqueous HCl and the reagent 2-iminothiolane in DMSO. To start labeling, Onc (50 μM at final) and 2-iminothiolane (1 mM at final) were mixed in the reaction buffer (50 mM phosphate and 150 mM NaCl; pH 8.0) and incubated at room temperature for 1 h. Thereafter, the reaction mixture was acidified to pH 3.0 by acetic acid and applied to gel filtration. The Onc fraction was eluted from a Sephadex^®^ G-25 column (Sinopharm) by 10% aqueous acetic acid, manually collected and lyophilized.

To prepare the CTX-Onc conjugate, the SPDP-labeled CTX and the 2-iminothiolane labeled Onc were dissolved in 1.0 mM aqueous HCl, respectively. To start conjugation, they were mixed together at equal concentrations (50 μM each) in the reaction buffer (50 mM NaPO_4_ and 150 mM NaCl; pH 8.0) and incubated at room temperature for 0.5 h. Thereafter, the reaction mixture was acidified to pH 3.0 by acetic acid and applied to gel filtration. The CTX-Onc conjugate was eluted from the Sephadex^®^ G-25 column by 10% acetic acid and lyophilized.

### Cytocoxicity of the CTX-Onc conjugate on the cultured glioma cells

Human glioma U251 and SHG-44 cells were purchased from the Cell Bank, Shanghai Institutes For Biological Sciences, Chinese Academy of Sciences (Shanghai, China) and cultured in Dulbecco’s modified Eagle’s medium supplemented with 10% fetal bovine serum and antibiotics, at 37°C in a CO_2_ incubator. For the cytotoxicity assay, the cells were seeded into a 96-well plate (5×10^3^ cells/well). The subsequent day, the cells were changed into the medium containing different concentrations of CTX-Onc conjugate or the physical mixture of CTX and Onc (CTX + Onc), and were continuously cultured for 36 h. Thereafter, the cell viability was assayed using the MTT method (MTT assay kiy; Solarbio, Beijing, China).

### Anti-glioma effect of the CTX-Onc conjugate on a nude mouse model

All animal experiments were approved by the Animal Care and Use Committee of Tongji University (Shanghai, China). The five-week-old male athymic mice (BALB/c) were obtained from Shanghai Laboratory Animal Center (Shanghai, China). The cultured U251 cells (5×10^7^ cells/mouse) were suspended in 200 μl phosphate-buffered saline (PBS) containing 50% Matrigel™ (BD Biosciences, Franklin Lakes, NJ, USA) and subcutaneously injected into the nude mice. The dimensions (length and width) of the tumors were measured by calipers, and the tumor burden was calculated using the following formula: 0.5 × length × width^2^. Once the tumor had grown to 200–300 mm^3^, it was removed and cut into uniform tablets (size, 10–20 mm^3^) subsequent to the mouse being sacrificed in a CO_2_ chamber, and then the tumor tablets (one tablet/mouse) were subcutaneously injected into the nude mice. The cultured SHG-44 cells (1×10^7^ cells/mouse) were suspended in 200 μl PBS and subcutaneously injected into the nude mice.

Two months after inoculation with the tablets, the nude mice bearing the U251 tumors were randomly divided into three groups (n=3 mice/group), and two of the groups were intravenously injected at 2.5 mg/kg with 0.2 ml CTX + Onc mixture dissolved in PBS and CTX-Onc conjugate dissolved in PBS, respectively, twice a week for ~1 month. The other group was intravenously injected with 0.2 ml PBS by the same method as a control. The nude mice bearing SHG-44 tumors were only divided into three groups (n=5 mice/group) two months after inoculation, and were then given the same treatment as the nude mice bearing the U251 tumors. The dimensions of the tumors and the weights of the mice were measured at least twice a week and recorded during the treatments.

## Results

### Chemical conjugation of the recombinant CTX and Onc

**Figure 1:**
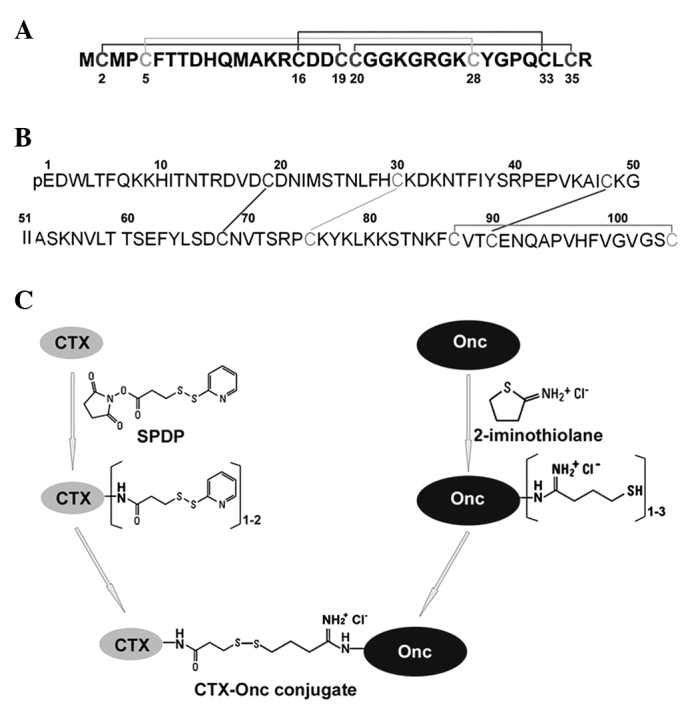
Amino acid sequence and disulfide linkages of (A) CTX and (B) Onc. (C) Strategy for preparation of the CTX-Onc conjugate in the present study. CTX, chlorotoxin; Onc, onconase.

As CTX can selectively bind malignant gliomas, the present study attempted to use it to target Onc toward gliomas. To prepare the CTX-Onc conjugate, an efficient conjugation procedure was established by which CTX and Onc were covalently crosslinked through reversible disulfide bond(s) (Fig. 1). Firstly, active disulfide(s) were introduced into the recombinant CTX by chemical modification using the primary amine-specific reagent SDPD, while free thiol(s) were introduced into the recombinant Onc by chemical modification using the primary amine-specific reagent 2-iminothiolane. Secondly, CTX-Onc conjugates were formed via reaction of the introduced free thiol(s) of the modified Onc with the introduced active disulfide bond(s) of the modified CTX. Owing to the presence of the reversible disulfide crosslinking in the present CTX-Onc conjugate, free Onc is likely to be released from the conjugate and to enter the glioma cells to exert its cytotoxicity, since the disulfide crosslinking may be reduced by the reductive redox potential at the cell membrane.

**Figure 2:**
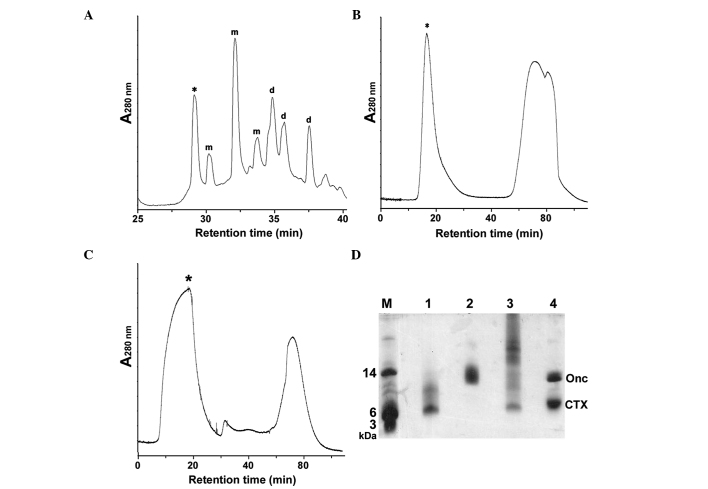
Preparation of the CTX-Onc conjugate. (A) Purification of the SPDP-modified CTXs by rp-HPLC. The peak of unlabeled CTX was indicated by *; the peaks of mono-labeled CTXs were indicated by the letter ‘m’; the peaks of di-labeled CTXs were indicated by the letter ‘d’. (B) Purification of the 2-iminothiolane-modified Onc by gel filtration. The first peak was the eluted Onc fraction (indicated by *) and the second peak was the excess modification reagent. (C) Purification of CTX-Onc conjugate by gel filtration. The first peak was the eluted CTX-Onc fraction (indicated by *) and the second peak was the salts in the reaction buffer. (D) Tricine SDS-PAGE analysis. Lane M, protein marker; lane 1, the SPDP-modified CTX; lane 2, the 2-iminothiolane-modified Onc; lane 3, CTX-Onc conjugate; lane 4, CTX-Onc conjugate treated by DTT. CTX, chlorotoxin; Onc, onconase; SPDP, N-Succinimidyl 3-(2-pyrdydithio)propionate; rp-HPLC, reverse phase high performance liquid chromatography; DTT, dithiothreitol.

CTX has four primary amine moieties, including one N-terminal α-amine and three internal ɛ-amines of the side-chain of lysine (Lys) residues (Fig. 1A). These primary amine moieties would all react with the primary amine-specific modification reagent SPDP during chemical modification. Thus, seven major peaks were eluted from the C18 reverse-phase column following SPDP modification, although the ratio of CTX to SPDP was carefully controlled (Fig. 2A). Mass spectrometry analyses showed that the first peak (indicated by a star) was the unlabeled CTX (measured molecular mass, 3,998.0; theoretical value, 3,996.8); peaks 2–4 were the mono-labeled CTXs (indicated by a letter ‘m’) carrying one active disulfide bond (measured molecular mass, 4,193.0; theoretical value, 4,194.8); peaks 5–7 were the di-labeled CTXs (indicated by a letter ‘d’) carrying two active disulfide bonds (measured molecular mass, 4,391.0; theoretical value, 4,391.8). In the subsequent conjugation reaction, the mono-labeled and di-labeled CTXs were mixed together and reacted with the modified Onc.

Although Onc has no N-terminal α-amine moiety due to the formation of a pyroglutamate residue, it has 12 internal ɛ-amine moieties of the side-chain of Lys residues (Fig. 1B). All these internal primary amine moieties would react with the reagent 2-iminothiolane, thus the reaction condition was optimized by controlling the ratio of Onc to 2-iminothiolane during the modification. Subsequently the excess 2-iminothiolane was removed by gel filtration (Fig. 2B) and the eluted Onc fraction was analyzed by mass spectrometry; 1–3 free thiol moieties were introduced into the majority of Onc molecules (measured molecular mass, 11,920.0, 12,022.0 or 12,121.0). In the subsequent conjugation reaction, the modified Onc mixture was used for conjugation with the SDPD-modified CTX.

To form the CTX-Onc conjugate, the SPDP-modified CTX and the 2-iminothiolane-modified Onc were mixed at a molar ratio of 1:1. Following the reaction, the CTX-Onc conjugate was eluted from a gel filtration column by 5% aqueous acetic acid (Fig. 2C) and analyzed by tricine SDS-PAGE (Fig. 2D). The CTX-Onc conjugate (lane 3) showed smear bands as it contained different numbers of CTX and Onc. Subsequent to treatment by the reducing reagent dithiothreitol, the conjugate (lane 4) showed two sharp bands corresponding to the modified CTX (lane 1) and the modified Onc, respectively. Thus, the conjugate was sensitive to the reducing condition and the Onc would be released from the conjugate under reducing conditions.

### Cytotoxicity of the CXT-Onc conjugate to the cultured glioma cells

**Figure 3:**
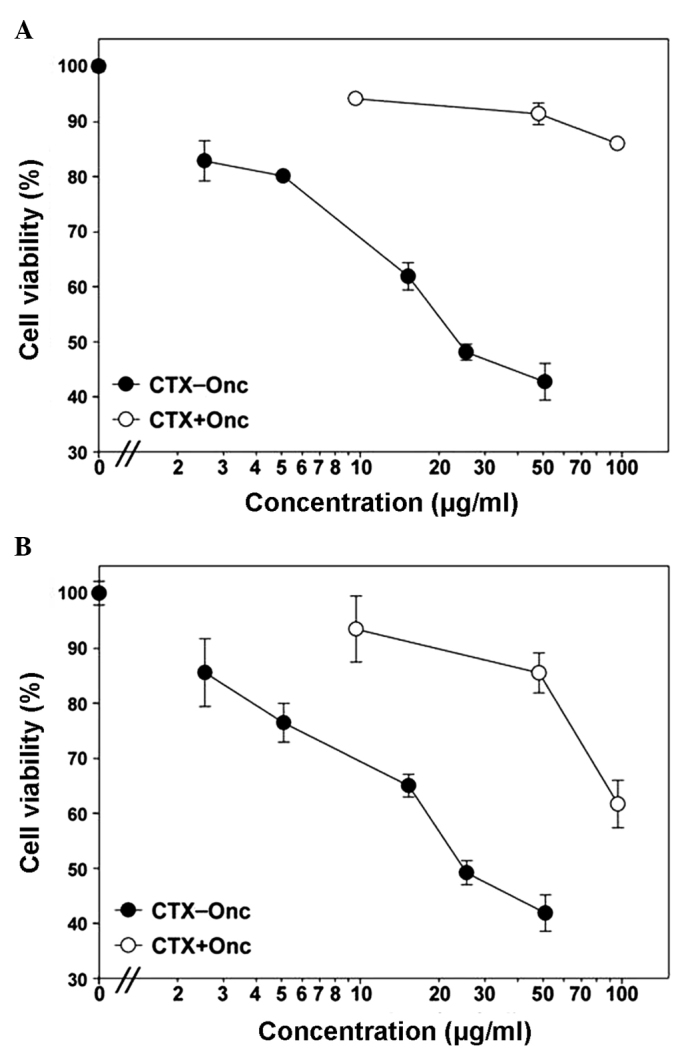
Cytotoxic effects of CTX-Onc conjugate and the CTX + Onc mixture on the cultured (A) U251 and (B) SHG-44 cells. CTX, chlorotoxin; Onc, onconase.

To test the anti-glioma effect of the CTX-Onc conjugate, its cytotoxicity to the cultured glioma cells was first measured. To test the targeting effect of CTX, the CTX + Onc mixture at equal molar ratio was used as a control, designated as CTX + Onc. Subsequent to treatment either by CTX-Onc conjugate or by CTX + Onc mixture, the viability of the glioma cells were measured by MTT assay. As shown in Fig. 3, the CTX-Onc conjugate had a dose-dependent cytotoxicity to the U251 and SHG-44 cells, with the concentration to inhibit cell survival to 50% (SF50) of ~20 μg/ml to the two cell types. Furthermore, the conjugate showed much higher (>10-fold) cytotoxicity than the CTX + Onc mixture, suggesting that CTX targeted more Onc to glioma cells and significantly enhanced its cytotoxicity.

### Anti-glioma effects of the CTX-Onc conjugate on a mouse model

**Figure 4:**
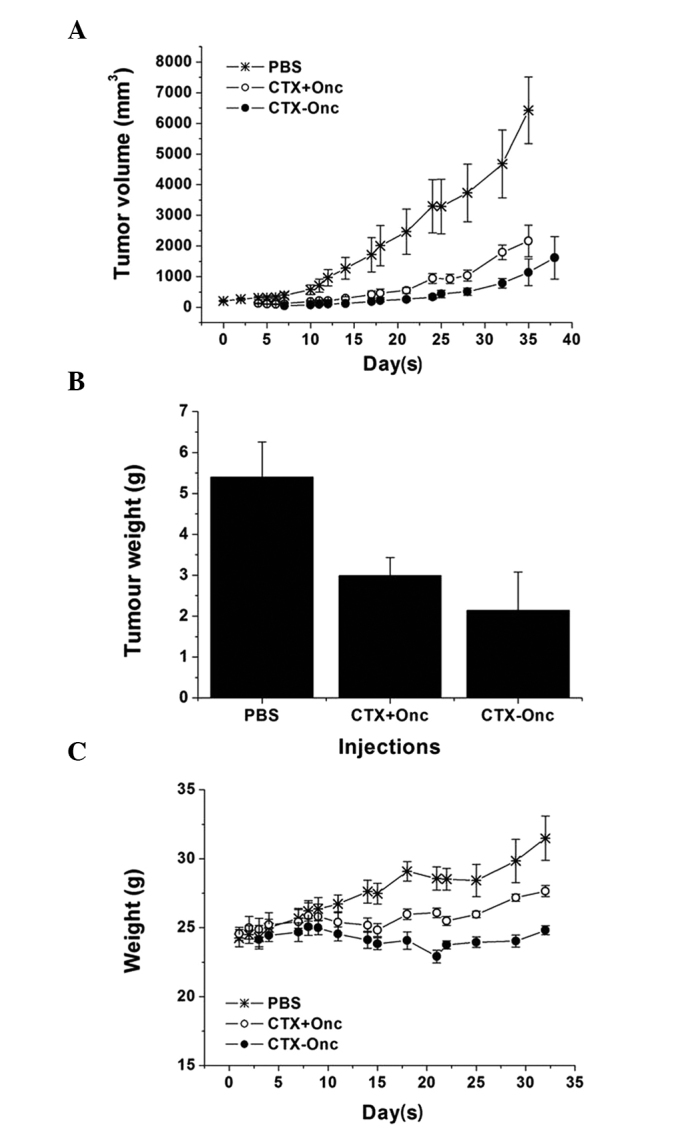
Anti-tumor effects of the CTX-Onc conjugate (2.5 mg/kg) and the CTX + Onc mixture (2.5 mg/kg) on the nude nice bearing U251 gliomas. (A) Tumors volumes are shown as a function of days during the treatment (bars indicate the mean volume of three tumors ± SE). (B) Final tumor weights are shown following the treatment (bars indicate the mean weight of three tumors ± SE). (C) Weights are shown as a function of days during the treatment (bars indicate the mean weights of three mice ± SE. SE, standard error; CTX, chlorotoxin; Onc, onconase; PBS, phosphate-buffered saline.

As the CTX-Onc conjugate had a significant anti-glioma effect on cultured cells, its effect was further tested on nude mice bearing either U251 or SHG-44 tumors. As shown in Fig. 4A, when the mice bearing subcutaneous U251 tumors were treated with CTX-Onc conjugate, CTX + Onc mixture or PBS for a month, the CTX-Onc conjugate showed the highest inhibition effect on the tumor growth, suggesting that CTX also had a targeting effect *in vivo.* Subsequent to the mice being sacrificed at the end of treatment, the mean tumor weight of the CTX-Onc group was also lower than that of the CTX + Onc group, confirming the CTX targeting effect (Fig. 4B).

**Figure 5:**
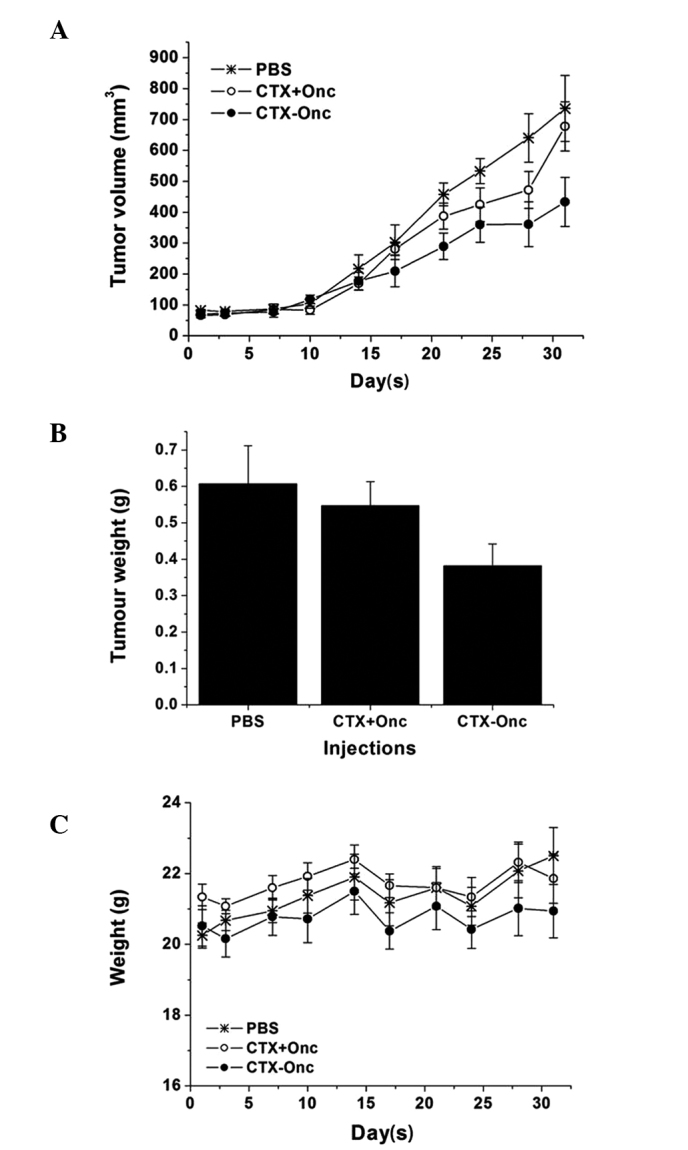
Anti-tumor effects of the CTX-Onc conjugate (2.5 mg/kg) and the CTX + Onc mixture (2.5 mg/kg) on the nude nice bearing SHG-44 gliomas. (A) Tumors volumes are shown as a function of days during the treatment (bars indicate the mean volume of five tumors ± SE). (B) Final tumor weights are shown following the treatment (bars indicate the mean weight of five tumors ± SE). (C) Weights are shown as a function of days during the treatment (bars indicate the mean weights of five mice ± SE). SE, standard error; CTX, chlorotoxin; Onc, onconase; PBS, phosphate-buffered saline.

The effect of the CTX-Onc conjugate on the mice bearing the subcutaneous SHG-44 tumors was also tested. After ~1 month of treatment, the CTX-Onc conjugate also showed the highest inhibition effect on tumor growth (Fig. 5A), and it also suggested that CTX had a targeting effect *in vivo*. Subsequent to the mice being sacrificed at the end of treatment, the mean tumor weight of the CTX-Onc group was also lower than that in the CTX + Onc group, confirming the CTX targeting effect again (Fig. 5B). As shown in Figs. 4C and 5C, the mean weights of the CTX-Onc group were not significantly affected during the treatment, suggesting that the CTX-Onc conjugate exhibited a low toxicity *in vivo*.

## Discussion

Malignant gliomas are common malignant tumors in the central nervous system. Due to the rapid proliferation and poor differentiation of gliomas, and the limited effects of the traditional treatments on gliomas, patients generally have a poor prognosis (2,3). Therefore, targeted therapy based on the identification of the tumor-specific target is deemed to be a promising approach for malignant glioma treatment. CTX can selectively bind malignant gliomas, and now TM-601 (I^131^-labeled CTX) has been used as a drug for gliomas in a clinical trial (36). However, the cytotoxicity of CTX is not strong enough to result in tumor cell apoptosis, therefore there was a requirement to link it with a molecule that can cause the tumor cell apoptosis. Onc exhibits anti-tumor effects on various tumors, with few side-effects, and has been used as a drug to treat malignant pleural mesothelioma (MPM) in a phase III clinical trial (37). Onc was selected to be linked to CTX in the preparation of an anti-glioma drug in the present study. There were two methods of preparation. One method was to construct the fusion gene of CTX and Onc, but the eight disulfide bonds contained by the fusion proteins of CTX and Onc may have resulted in difficulty in refolding of the fusion protein *in vitro.* Therefore the other method of preparation was selected. The recombinant CTX and Onc were respectively obtained and linked by a disulfide bond to prepare the CTX-Onc conjugate, rather than use of the chemical linkage, which was always used in preparation for the monoclonal antibody-targeted drugs and can be broken in the lysosome. Since it was not clear whether the membrane protein, particularly MMP-2, on the glioma cell surface bound by CTX could enter the cells by endocytosis, in order to prepare the CTX-Onc conjugate, a reversible chemical crosslinking approach was established. By reaction with the primary amine-specific bifunctional reagent SPDP, active disulfide bond(s) were introduced into the recombinant CTX; by reaction with the primary amine-specific reagent 2-iminothiolane, free thiol group(s) were introduced into the recombinant Onc. Via reaction of free thiol(s) of the modified Onc with active disulfide bond(s) of the modified CTX, the CTX-Onc conjugate was obtained. It was expected that the disulfide bond between CTX and Onc could be broken by the reducing power on the cell surface when the CTX-Onc conjugate bound to the glioma cells by CTX, and that the Onc released could enter cells by electrostatic interaction and endocytosis to degrade RNA in the glioma cells.

The results of the *in vitro* and *in vivo* experiments demonstrated that the CTX-Onc conjugate exhibited an improved anti-tumor effect compared with the CTX + Onc mixture, suggesting that CTX could target more Onc to glioma cells and significantly enhance its cytotoxicity. The anti-tumor effect of CTX-Onc is similar to that of the CTX-conjugated nanoparticle (NP-MTX-CTX) reported by Sun *et al in vitro*, which was capable of inhibiting >50% of glioma cell survival at ~100 μg/ml (14). Furthermore, the anti-tumor effect of the CTX-Onc conjugate is greater than that of the CTX-based conjugate (ClTx-LS) reported by Xiang *et al in vivo*, which had an inhibition ratio of ~34% compared that of the saline group, at 5 mg/kg (38). When compared with the inhibition ratio of the CTX + Onc mixture on tumor growth *in vivo*, the ratio of the CTX-Onc conjugate increased by 15–30%. In addition, it was revealed that the injection method could impact on the inhibitory effect of the CTX-Onc conjugate on gliomas *in vivo*. The intraperitoneal injection with CTX-Onc conjugate did not result in any inhibitory effect on the growth of the glioma. This may be associated with the large molecular weight of the CTX-Onc conjugate, which is prevented from entering the blood system, or with the fact that the CTX-Onc conjugate may be digested by proteases in the peritoneal cavity. Finally, intravenous injection was selected as the method of drug delivery. Although the orthotopic glioma model *in vivo* was not used to determinate the anti-tumor effect of the CTX-Onc conjugate in the present study, the *in vivo* results can be used as the basis for the future clinical study of the CTX-Onc conjugate, since the drug delivery in the treatment of gliomas in clinical practice can be made through the cerebrospinal fluid to avoid the blood-brain barrier. Therefore, the CTX-Onc conjugate designed in the present study can be used as a novel potential anti-glioma drug for further studies.
